# Clitorin ameliorates western diet-induced hepatic steatosis by regulating lipogenesis and fatty acid oxidation in vivo and in vitro

**DOI:** 10.1038/s41598-022-07937-3

**Published:** 2022-03-09

**Authors:** Divina C. Cominguez, Yea-Jin Park, Yun-Mi Kang, Agung Nugroho, Suhyun Kim, Hyo-Jin An

**Affiliations:** 1 Department of Pharmacology, College of Korean Medicine, Sangji University, Wonju, Gangwon-do 26339 Republic of Korea; 2 Department of Agro-Industrial Technology, Lambung Mangkurat University, Banjarbaru, Indonesia; 3 Department of Obstetrics & Gynecology College of Korean Medicine, Sangji University, Wonju-si, Gangwon-do 26339 Republic of Korea

**Keywords:** Cell biology, Plant sciences, Diseases

## Abstract

Nonalcoholic fatty liver disease (NAFLD) is usually correlated with metabolic diseases, such as obesity, insulin resistance, and hyperglycemia. Herein, we investigated the inhibitory effects and underlying governing mechanism of clitorin in a western diet (WD)-induced hepatic steatosis mouse model, and in oleic acid-stimulated HepG2 cells. Male C57BL/6 mice were fed a normal diet, WD, WD + 10 or 20 mg/kg orlistat, and WD + 10 or 20 mg/kg clitorin. HepG2 cells were treated with 1 mM oleic acid to induce lipid accumulation with or without clitorin. Clitorin significantly alleviated body weight gain and hepatic steatosis features (NAFLD activity score, micro-, and macro-vesicular steatosis) in WD-induced hepatic steatosis mice. Additionally, clitorin significantly decreased protein expressions of sterol regulatory element-binding protein 1 (SREBP1), peroxisome proliferator-activated receptor γ (PPARγ), and CCAAT/enhancer binding protein α (C/EBPα) in WD-induced hepatic steatosis mice. Moreover, clitorin significantly diminished the mRNA levels of *SREBP1*, acetyl-CoA carboxylase (*ACC*), fatty acid synthase (*FAS*), and hydroxy-3-methylglutaryl coenzyme A reductase (*HMGCR*) and enhanced the mRNA levels of peroxisome proliferator-activated receptor α (*PPARα*) and carnitine palmitoyltranserase-1 (*CTP-1*), as well as adenosine monophosphate-activated protein kinase (*AMPK*) in the liver of WD-induced hepatic steatosis mice and oleic acid-stimulated HepG2 cells. Overall, our findings demonstrated that clitorin can be a potentially efficacious candidate for NAFLD management.

## Introduction

Nonalcoholic fatty liver disease (NAFLD) is the most ubiquitous chronic liver disease in Western countries, affecting nearly 25% of adults worldwide^1^. In the United States, the number of NAFLD cases is anticipated to increase from 83.1 million in 2015 to 100.9 million in 2030^2^. NAFLD ranges from relatively benign nonalcoholic fatty liver to the aggressive form termed nonalcoholic steatohepatitis, typifying both fatty liver and liver inflammation^3^. NAFLD is usually correlated with metabolic diseases, such as obesity, insulin resistance, hyperglycemia, and hypertension. Although considerable progress has been achieved with regard to drug development for NAFLD, no suitable therapeutic agent has yet been approved^2^. Therefore, there is a critical need to develop optimal therapeutic agents for NAFLD.

Under normal condition, liver processes large quantities of fatty acid, but stores only small amounts in the form of triglyceride^4^. Overnutrition directly contributes to the abundance of hepatic triglyceride accumulation, which lead to NAFLD progression^5^; herein, the main causes of hepatic steatosis are increased de novo lipogenesis and decreased fatty acid oxidation^6^. Many genes play important roles in lipogenesis and fatty acid oxidation in the liver. When the high-fat diet feeding, peroxisome proliferator-activated receptor γ (PPARγ) is the early-induced lipogenic transcription factor^7^. Liver X receptor (LXR) and sterol regulatory element–binding protein 1c (SREBP1c) are key transcription factors involved in hepatic lipid synthesis^7^. Fatty acid synthase (FAS) and 3-hydroxy-3-methylglutaryl coenzyme A reductase (HMGCR) are key enzymes in the synthesis of fatty acid and cholesterol, respectively^8^. Acetyl-CoA carboxylase (ACC) catalyzes a master rate-controlling step in de novo lipogenesis and fatty acid oxidation^9,10^. Peroxisome proliferator-activated receptor α (PPARα) is closely associated with the transcription of genes related to hepatic fatty acid oxidation, including carnitine palmitoyltransferase-1 (CPT-1)^11^.

Adenosine monophosphate-activated protein kinase (AMPK) is master regulator for energy homeostasis through the inhibition of lipogenesis and the activation of fatty acid oxidation in liver^12^. Although multiple factors lead to hepatic steatosis, to control lipogenesis and fatty acid oxidation can be therapeutic strategies for management of NAFLD individuals who consume excess calories^4^.

Papaya (*Carica papaya* L.) is a fruit crop that is widely grown in tropical and sub-tropical regions. Traditionally, papaya plants are used to treat various ailments such as asthma, ulcers, eczema, diabetes, helminth infections, and fever^13^. Papaya plants have been reported to possess therapeutic potential for metabolic disorders, such as diabetes mellitus type 2, causing alterations in both glycemic metabolism and lipid metabolism, oxidative stress, and in models of arterial hypertension^13,14^. Previous profiling indicates that four flavonoids, including manghaslin, clitorin, rutin, and nicotiflorin, were identified in papaya plants^15,16^. Among them, we focused on clitorin, a kaempferol glycoside, because it has only been reported antioxidant effects^17^. Based on these findings, the present study was designed to provide basic data to delineate the pharmacological effects of clitorin on the alleviation of hepatic steatosis in western diet (WD)-induced hepatic steatosis mice and oleic acid-stimulated HepG2 cells.

## Results

### Clitorin reduced the body weight and liver weight index in the WD-fed obese mice

**Figure 1:**
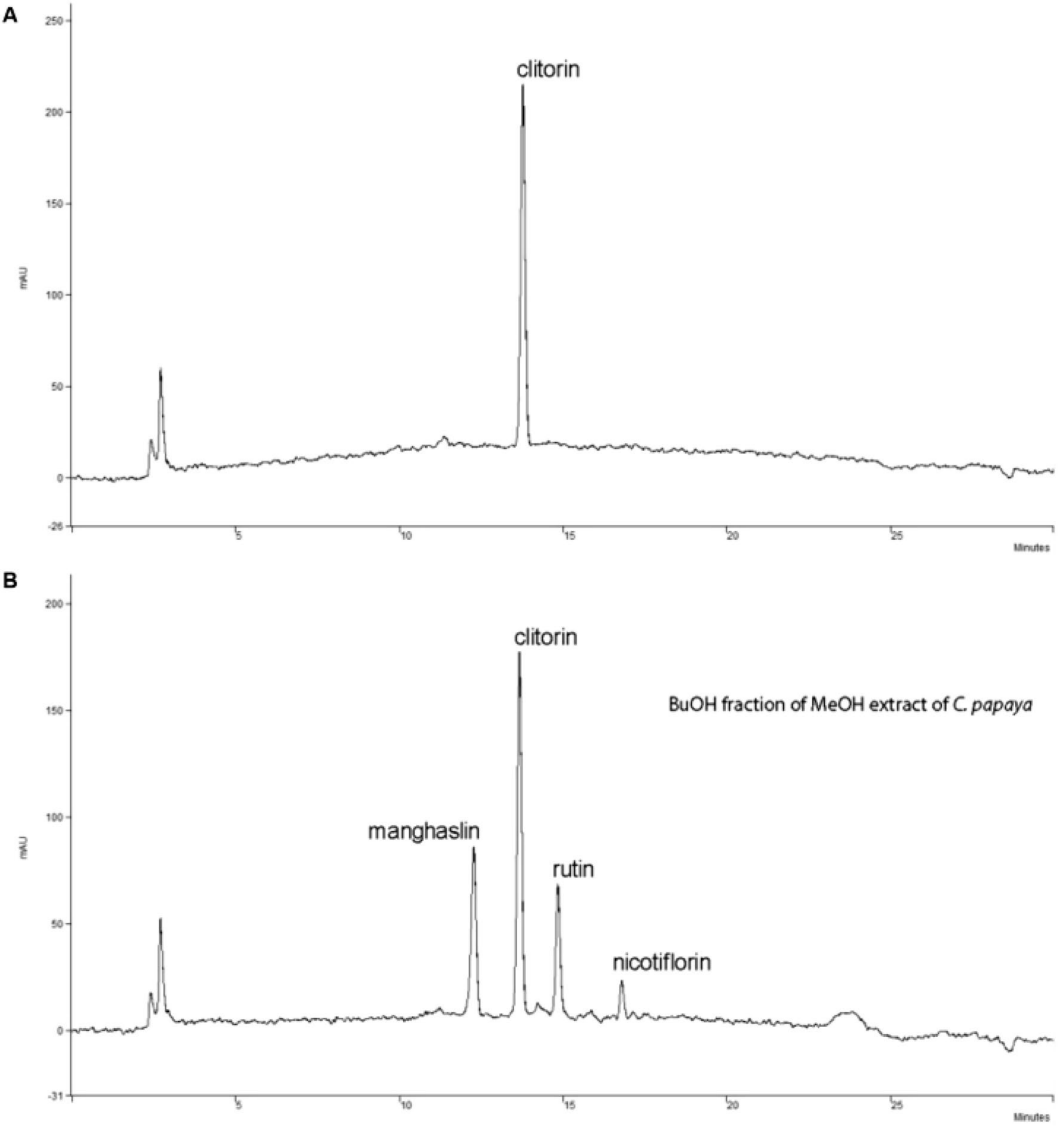
Isolation and identification of clitorin. HPLC chromatograms of (**A**) clitorin and (**B**) papaya plants. A peak arose before clitorin was manghaslin, and two peaks arose after clitorin were rutin and nicotiflorin, consecutively. HPLC: high performance liquid chromatography.

**Figure 2:**
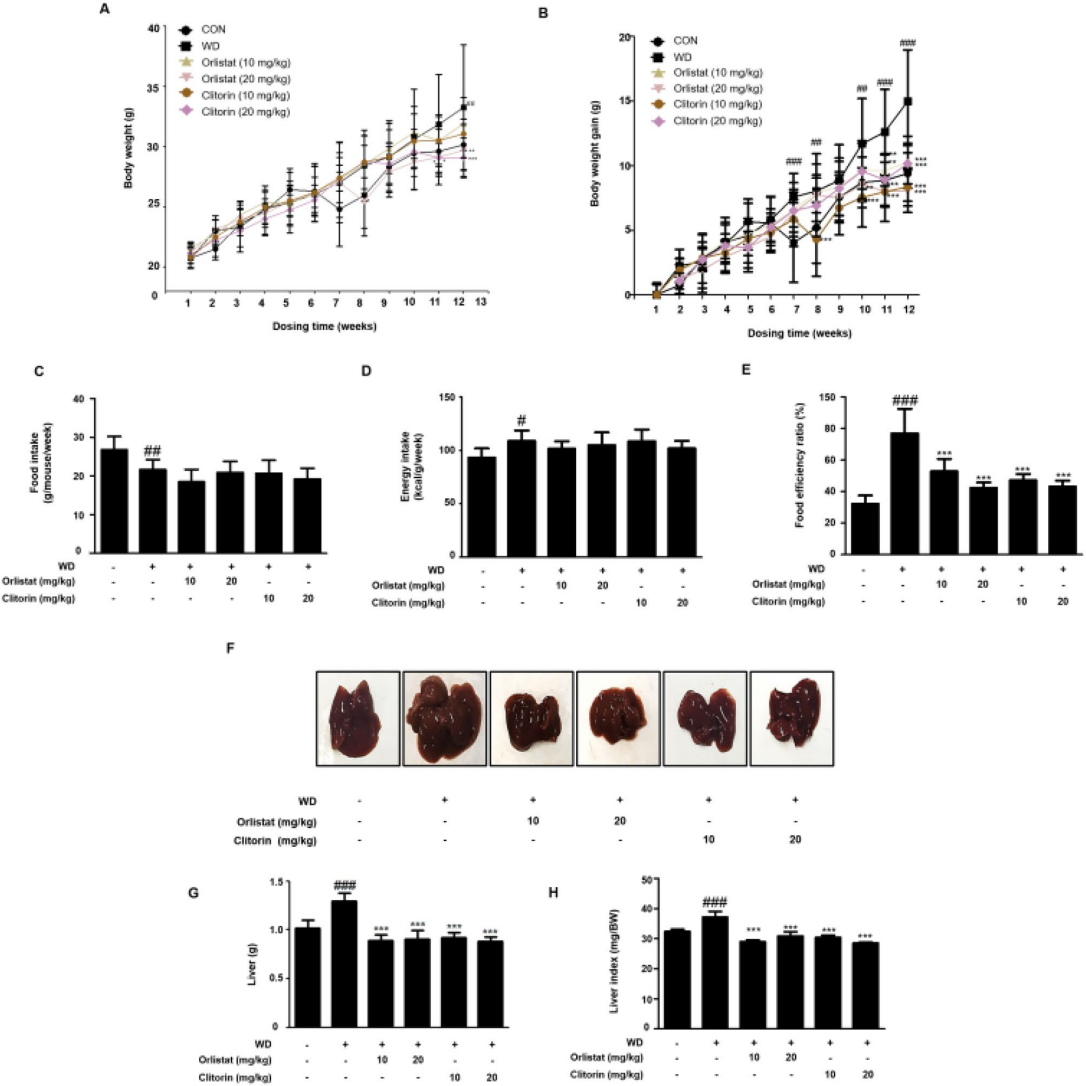
Effect of clitorin on total body weight, weight gain, and food intake in the WD-induced obese mouse model. The WD-induced mice were administered orlistat (10 or 20 mg/kg) or clitorin (10 or 20 mg/kg) for 4 weeks, whereas control mice were fed a normal diet. (**A**) Body weight and (**B**) weight gain were recorded every week. (**C**) Food intake and (**D**) energy intake were calculated. (**E**) Food efficiency ratio (FER) was calculated by applying the equation: FER = (body weight gain (g)/food intake (g)) × 100. (**F**) Macroscopic images in the liver of mice in each group were taken at the end of the 13-week experimental period. (**G**) The weight of liver tissue and (**H**) relative liver weight ratio (mg/body weight) were measured. The values are represented as the mean ± SD (n = 6 per group). ^#^P < 0.05, ^##^P < 0.01, and ^###^P < 0.001 vs. CON group; *P < 0.05, **P < 0.01, and ***P < 0.001 vs. WD group; significance was determined using two-way ANOVA followed by a Bonferroni post hoc test, and one-way ANOVA followed by Dunnett’s post hoc test. *WD* western diet, *FER* food efficiency ratio.

We have isolated the clitorin from papaya plants, identified by high performance liquid chromatography (HPLC) analysis (Fig. 1), and investigated the effect of clitorin on the WD-fed obese mice. When mice were fed a WD for 12 weeks, we observed significant differences on total body weight and weight gain between the CON and WD groups. However, the orlistat- or clitorin-administered groups displayed significantly lower body weight and weight gain than the WD group (Fig. 2A,B). Herein, we did not observe any differences in food intake and energy intake among all WD-fed groups (Fig. 2C,D), while the food efficiency ratio was significantly attenuated after orlistat and clitorin administration in the WD-induced obese mice (Fig. 2E). In addition, we found that the liver weight and liver index (mg/body weight) in the WD group were significantly higher than the corresponding parameters in the CON group, indicating that the hepatic steatosis was induced in the WD-fed obese mice. Notably, orlistat and clitorin administration significantly reversed these changes compared to those observed in the WD group (Fig. 2F–H).

### Clitorin ameliorated the features of liver steatosis in the WD-induced hepatic steatosis mice

**Figure 3:**
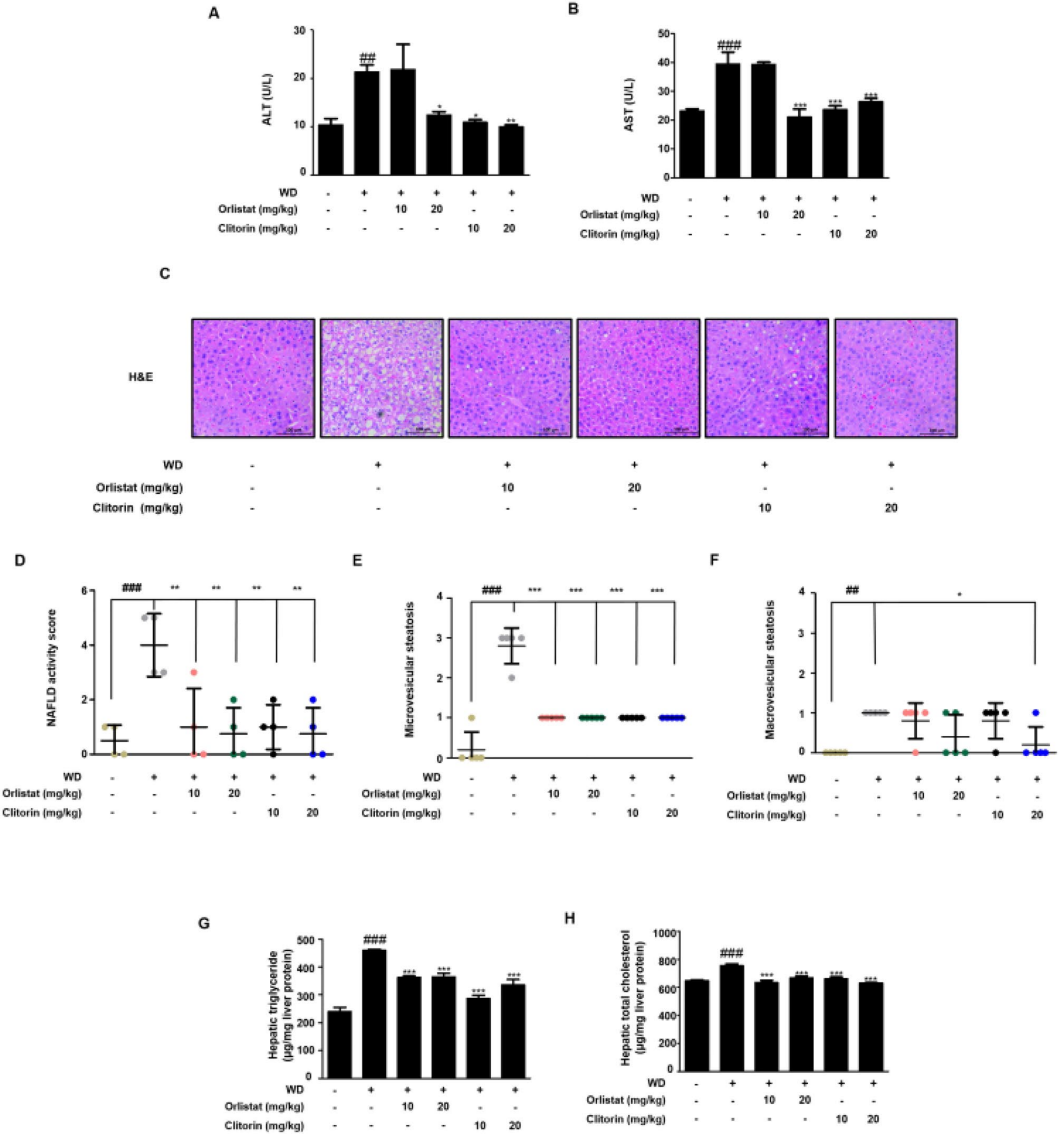
Effect of clitorin on the hepatic steatosis in the WD-induced hepatic steatosis mouse model. The levels of serum (**A**) ALT and (**B**) AST were determined using enzymatic methods. (**C**) The liver tissues from representative mice in each group were fixed, embedded in paraffin, and stained with H&E solution. Images are shown at an original magnification of 200 × . The scale bar is 100 µm. (**D**) NAS, (**E**) micro-, and (**F**) macro-vesicular steatosis were determined. The levels of hepatic (**G**) triglyceride and (**H**) total cholesterol were determined using enzymatic methods. The values are represented as the mean ± SD (n = 6 per group). ^##^P < 0.01 and ^###^P < 0.001 vs. CON group; *P < 0.05, **P < 0.01, and ***P < 0.001 vs. WD group; significance was determined using one-way ANOVA followed by Dunnett’s post hoc test. *WD* western diet, *ALT* alanine aminotransferase, *AST* aspartate aminotransferase, *H&E* hematoxylin and eosin.

Next, we checked the serum alanine aminotransferase (ALT) and aspartate aminotransferase (AST) levels. The significantly increased serum levels of ALT and AST by HFD feeding were effectively decreased in the 20 mg/kg orlistat-, and all clitorin-administered groups (Fig. 3A,B). To further confirm the increase in liver weight and liver index in the WD-induced obese mice model, we conducted hematoxylin and eosin (H&E) staining to evaluate the histological changes in the liver. We observed hepatocytic lipid vacuoles and hepatocyte ballooning in the WD group compared to those in the CON group; however, the orlistat- or clitorin-administered groups remarkably reversed the corresponding features in the liver of WD-fed obese mice (Fig. 3C). In addition, the significantly increased NAFLD activity score (NAS), micro-, and macro-vesicular steatosis were displayed in the WD group, whereas NAS and microvesicular steatosis were improved after orlistat or clitorin administration (Fig. 3D,E); notably, the macrovesicular steatosis was significantly reduced in the only clitorin 20 mg/kg-administered group (Fig. 3F). Furthermore, the marked increases of intrahepatic triglyceride and total cholesterol levels in the WD group were all lower in the orlistat- or clitorin-administered groups (Fig. 3G,H). Altogether, these data indicated that WD-induced hepatic steatosis mouse model was completely established and clitorin administration demonstrated a protective effect against hepatic steatosis in mice challenged with WD.

### Clitorin regulated adipogenesis, lipogenesis, and fatty acid oxidation in the liver of WD-induced hepatic steatosis mice

**Figure 4:**
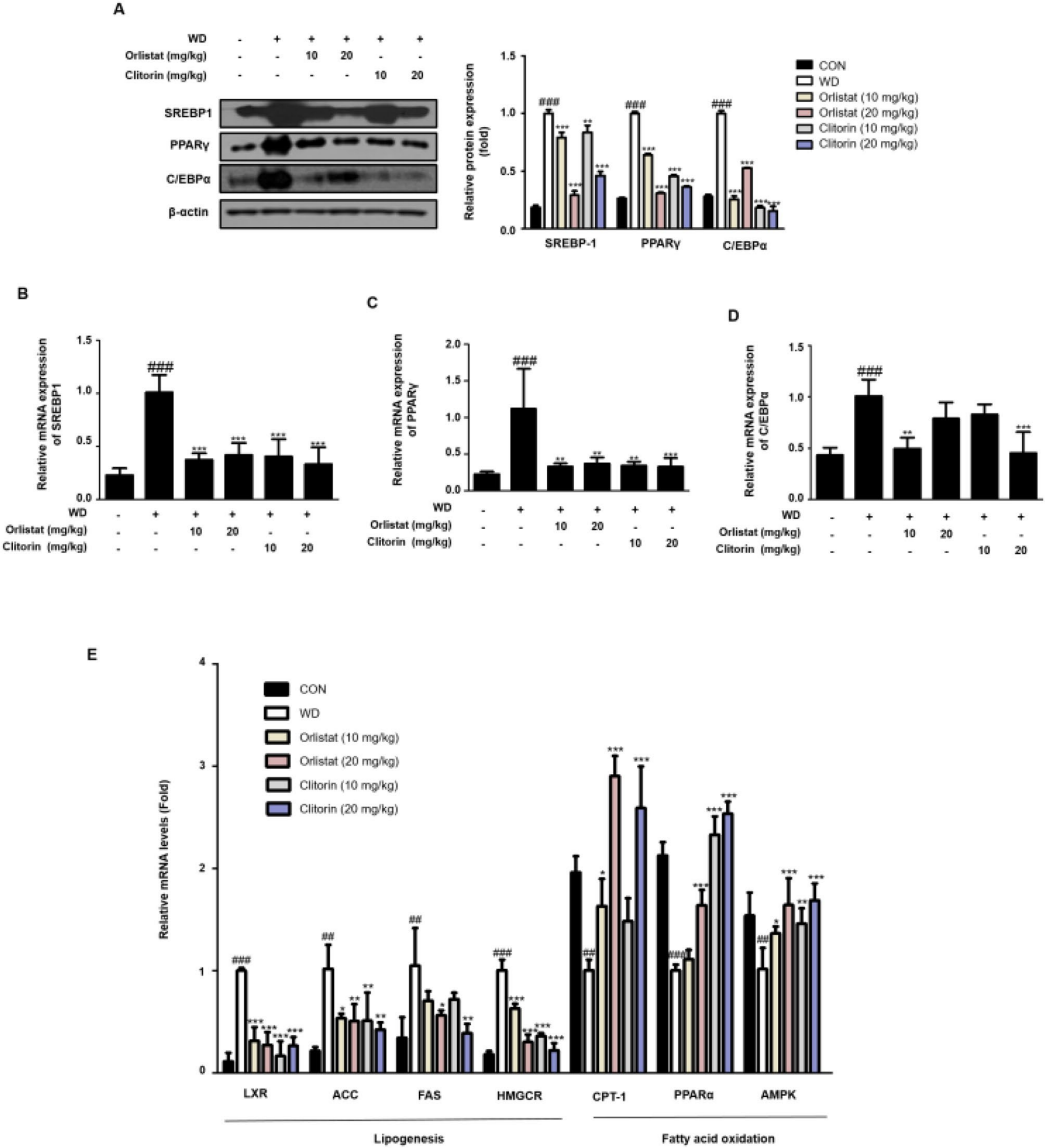
Effect of clitorin on adipogenic, lipogenic, and fatty acid oxidation-related genes in the liver of WD-induced hepatic steatosis mouse model. The protein levels of (**A**) SREBP1, PPARγ, and C/EBPα were determined using western blot analysis. The cropped gel images are shown for clarity. Densitometric analysis was performed using ImageJ ver. 1.50i (https://imagej.nih.gov/ij/). The mRNA levels of (**B**) *SREBP1*, (**C**) *PPARγ*, (**D**) *C/EBPα*, and (**E**) *LXR*, *ACC*, *FAS, HMGCR, CPT-1*, *PPARα*, and *AMPK* were determined using qRT-PCR analysis. The values are represented as the mean ± SD (n = 6 per group). ^#^P < 0.05, ^##^P < 0.01, and ^###^P < 0.001 vs. CON group; *P < 0.05, **P < 0.01, and ***P < 0.001 vs. WD group; significance was determined using one-way ANOVA followed by Dunnett’s post hoc test. *WD* western diet, *SREBP1* sterol regulatory element binding protein 1, *PPARγ* peroxisome proliferator activated receptor γ, *C/EBPα* CCAAT/enhancer binding protein α, *LXR* liver X receptor, *ACC* acetyl-CoA carboxylase, *FAS* fatty acid synthase, *HMGCR* 3-Hydroxy-3-Methylglutaryl-CoA Reductase, *CPT-1* Carnitine palmitoyltransferase-1, *PPARα* peroxisome proliferator activated receptor α, *AMPK* adenosine monophosphate-activated protein kinase.

To investigate the mechanisms of clitorin on suppressing hepatic steatosis in the WD-induced hepatic steatosis mice, adipogenic and lipogenic transcriptional expression profiles were examined. In the liver, the protein expression levels of SREBP1, as well as PPARγ and C/EBPα, were higher in the WD group; however, this was rescued by orlistat and clitorin administration (Fig. 4A). qRT-PCR analysis revealed that the upregulated SREBP1, PPARγ, and C/EBPα protein expression levels in the livers of WD mice coincided with increases in *SREBP1*, *PPARγ*, and *C/EBPα* mRNA levels. Notably, the marked upregulation of the mRNA levels of *SREBP1* and *PPARγ* was strongly suppressed in the liver of orlistat- or clitorin-administered mice compared to the corresponding expression profiles in the WD group (Fig. 4B,C). The mRNA level of *C/EBPα* was significantly down-regulated in the 10 mg/kg orlistat- or 20 mg/kg clitorin-administered mice; herein, 10 mg/kg orlistat-administered group had stronger inhibitory effect than the 20 mg/kg orlistat-administered group in C/EBP*α* protein expression (Fig. 4A), and this tendency was also observed in the mRNA level of C/EBP*α* (Fig. 4D). We next assessed the impact of clitorin on the mRNA levels of lipogenesis (*LXR*, *ACC*, *FAS*, and *HMGCR*) and fatty acid oxidation genes (*CPT-1*, *PPARα*, and *AMPK*), and it was revealed that the mRNA levels of *LXR* and *HMGCR* were significantly inhibited in the all orlistat- or clitorin-administered groups (Fig. 4E). In addition, the mRNA levels of *ACC* and *FAS* were significantly repressed in the 20 mg/kg orlistat- or clitorin- administered groups, showing higher reduction in the clitorin-administered group than those in the orlistat-administered group (Fig. 4E). In addition, *CPT-1* mRNA level was significantly up-regulated in the 20 mg/kg orlistat- or clitorin-administered mice. *PPARα* and *AMPK* mRNA levels, which was eliminated in the WD group, were significantly augmented by 20 mg/kg orlistat- or all dose of clitorin-administered groups; additionally, these mRNA levels were higher after the clitorin administration than those in the orlistat-administered group (Fig. 4E). These data supported the concept that clitorin alleviates hepatic steatosis by regulating lipogenesis and fatty acid oxidation genes in the liver of WD-induced hepatic steatosis mice.

### Clitorin suppresses oleic acid-induced lipid accumulation in HepG2 Cells

**Figure 5:**
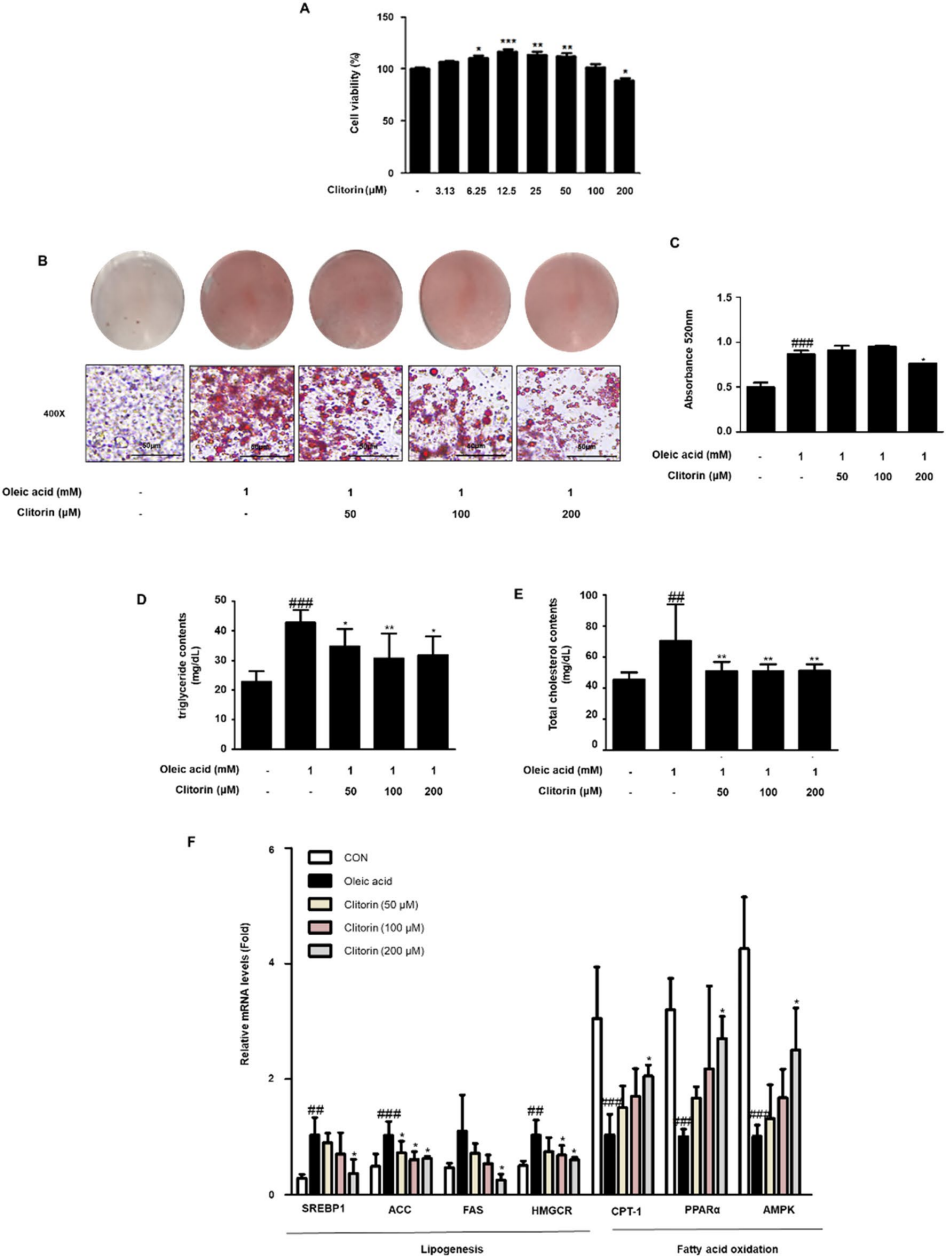
Effect of clitorin on oleic acid-induced lipid accumulation in HepG2 Cells. (**A**) Cell viability was evaluated in HepG2 cells treated with clitorin. (**B**) The lipid accumulation was determined by Oil Red O staining. Images are shown at an original magnification of 400 × . The scale bar is 50 µm. (**C**) The lipid content was quantified by measuring absorbance. The levels of secreted (**D**) triglyceride and (**E**) total cholesterol in HepG2 cells were determined using enzymatic methods. The mRNA levels of (**F**) *SREBP1*, *ACC*, *FAS, HMGCR, CPT-1*, *PPARα*, and *AMPK* were determined using qRT-PCR analysis. The values are represented as mean ± S.D of three independent experiments. ^##^P < 0.01 and ^###^P < 0.001 vs. non-treated cells; *P < 0.05, **P < 0.01, and ***P < 0.001 vs. oleic acid-treated cells; significances were determined using one-way ANOVA followed by Dunnett’s post hoc test. *SREBP1* sterol regulatory element binding protein 1, *ACC* acetyl-CoA carboxylase, *FAS* fatty acid synthase, *HMGCR* 3-Hydroxy-3-Methylglutaryl-CoA Reductase, *CPT-1* Carnitine palmitoyltransferase-1, *PPARα* peroxisome proliferator activated receptor α, *AMPK* adenosine monophosphate-activated protein kinase.

To further eliminate the effect of clitorin on lipid metabolism, relevant experiments were performed in vitro with HepG2 cells. The MTT assay showed that clitorin treatment (0 − 200 μM) did not induce cytotoxicity in HepG2 cells for 24 h (Fig. 5A). Accordingly, we designated three doses of clitorin at 50, 100, and 200 µM for further study. Oil Red O staining exhibited an obvious increase in stained lipid droplets in oleic acid-stimulated HepG2 cells compared to that in the non-treated cells, whereas it was significantly decreased after 200 µM clitorin treatment (Fig. 5B,C). Moreover, clitorin treatment significantly repressed the increased contents of triglyceride and total cholesterol in the oleic acid-stimulated HepG2 cells (Fig. 5D,E). Furthermore, the mRNA levels *SREBP1*, *FAS*, *ACC*, and *HMGCR* were higher in the oleic acid-treated group than those in the non-treated group; however, this was strongly reversed by 200 µM clitorin treatment (Fig. 5F). Moreover, *CPT-1*, *PPARα*, and *AMPK* mRNA levels, which was significantly down-regulated in the WD group, were effectively reversed by 200 µM clitorin treatment (Fig. 5F). These data indicated that clitorin attenuates oleic acid-induced hepatic lipid accumulation via control of the lipogenesis and the fatty acid oxidation.

## Discussion

Immoderate exposure to a high-fat diet has been determined as a key attribute in an increasing number of NAFLD patients, which is demonstrated by the fact that the prevalence of NAFLD in obese patients is reported to be up to 90%^18^; consequently, a high-fat diet is widely used to construct NAFLD animal models^19^. In the present study, clitorin administration significantly reduced the body weight gain in the WD-induced obese mice (Fig. 2A,B). To evaluate whether the hepatic steatosis was induced in this model, we first investigated the liver weight and index (mg/body weight), and the data showed that WD feeding caused the remarkable increases in the corresponding parameters compared to those in the CON group; on the other hand, clitorin administration significantly reduced the liver weight and index in the WD-induced obese mice (Fig. 2G,H). It has been reported that orlistat effectively alleviates steatosis and may serve as a viable treatment option for NAFLD^20^, therefore, it was used as a positive control. Next, we confirmed that clitorin administration significantly rescued the markedly increased serum levels of ALT and AST in WD-fed obese mice (Fig. 3A,B). Even though NAFLD typically is characterized by mild increases of serum ALT and AST, the normal ALT and AST levels can be observed in up to 50% of NAFLD patients^21^. In addition, previous clinical review study suggested that relying on liver enzyme abnormalities is unhelpful in the diagnosis of NAFLD and examination of disease severity^22^. For assessment of hepatic steatosis, the NAS, which includes steatosis, lobular inflammation, and ballooning, has been used in numerous clinical trials and cross-sectional studies^23^. Therefore, we conducted the H&E staining to further track whether the hepatic histological changes were induced in this in vivo model. We found that the NAS, micro-, and macro-vesicular steatosis were all higher in the WD-fed mice than the normal diet-fed mice; moreover, the marked elevations in the hepatic triglyceride and total cholesterol contents were displayed in the WD group, indicating that the WD-induced hepatic steatosis mouse model was completely established. Furthermore, clitorin administration significantly recovered the NAS and microvesicular steatosis (Fig. 3D,E) as well as lipid profiling (Fig. 3G,H) compared to those in the WD group. Notably, our data revealed that clitorin had stronger effect in suppressing macrovesicular steatosis than the orlistat (Fig. 3F).

The hepatic effect of PPARγ appears to be steatogenic; hepatocyte-specific *PPARγ* knockout mice showed a remarkable decrease in the number of hepatic lipid vacuoles, as well as downregulation of de novo lipogenesis activators^24^. Conversely, PPARγ overexpression in the liver induced by HFD feeding leads to lipid accumulation, which is the initiation step in the development of NAFLD^7^. CCAAT/enhancer binding proteins (C/EBPs), including C/EBPα and SREBP1, are also considered key regulators of adipogenesis. SREBP1 plays an important role in the regulation of de novo lipogenesis in the liver^25^. SREBP1c levels are enhanced in the fatty livers of obese, insulin-resistant, and hyperinsulinemic *ob*/*ob* mice^10^. In addition, SREBP1c expression is also elevated in patients with NAFLD; additionally, in concordance with its lipogenic role, hepatic triglyceride levels are higher in SREBP1c-overexpressing transgenic mice^26^. Thus, SREBP1, PPARγ, and C/EBPα are crucial transcription factors that upregulate the expression of genes modulating fat accumulation in the liver. In this study, the hepatic mRNA and protein levels of SREBP1, PPARγ, and C/EBPα were significantly reduced after clitorin administration in a dose-dependent manner (Fig. 4A–D). However, both protein and mRNA levels of C/EBPα were effectively abolished in the 10 mg/kg orlistat-administered mice than those in the 20 mg/kg orlistat-administered mice. Additionally, in the orlistat-administrated groups, the mRNA levels of SREBP1 and PPARγ were not consistent with the protein expressions; 10 mg/kg orlistat-administered group had higher inhibitory effects in the mRNA levels of SREBP1 and PPARγ than those in the 20 mg/kg orlistat-administered group. This probably seems to have relatively limitation in our study, with only 6 mice in each group examined in both protein and mRNA expression; therefore, further studies (e.g. increase ‘n’ per a group) would be examined to prove these differences.

LXRs are involved in hepatic lipogenesis via direct regulation of SREBP1c^27^, which positively modulates ACC expression^28^. ACC catalyzes a key rate-limiting step in fatty acid biosynthesis, and is also associated with the control of fatty acid oxidation by the synthesis of malonyl-CoA, an inhibitor of CPT-1^6^. Indeed, inhibition of the liver-specific isoform ACC1 in mice ameliorated hepatic triglyceride levels in mice by simultaneously suppressing fatty acid biosynthesis and augmenting fatty acid beta oxidation in the liver^6^. CPT-1 leads to beta-oxidation, as it allows fatty acids to reach the mitochondrial matrix^11^. CPT-1 is also linked to PPARα expression. Among the three PPAR isotypes, PPARα, PPARβ/δ, and PPARγ, PPARα is the most abundant isotype in hepatocytes and is related to numerous aspects of lipid metabolism^29^ and high fatty acid oxidation rates^30^. Ineffective PPARα sensing leads to diminished energy burning, resulting in hepatic steatosis and steatohepatitis^31^; thus, it is inferred that these genes can potentially prevent NAFLD. AMPK, a major energy sensor of the cell, downregulates ACC activity to suppress lipid biosynthesis^32^. In addition, AMPK regulates hepatic and adipose lipid metabolism by modulating lipogenesis, lipolysis, gluconeogenesis, and adipogenesis. AMPK inhibits de novo lipogenesis by downregulating *PPARγ*, *C/EBPα*, and *SREBP1*; furthermore, it promotes fatty acid oxidation by upregulating *CPT-1a*^33^. Our results showed that clitorin administration significantly decreased the mRNA levels of *LXR*, *ACC*, *FAS*, and *HMGCR*, which are lipogenic genes, and it also enhanced the mRNA levels of *PPARα* and *CTP-1*, fatty acid oxidation genes, as well as *AMPK* mRNA levels in the livers of WD-induced hepatic steatosis mice (Fig. 4E). Overall, clitorin had similar or excellent inhibitory effects compared to orlistat used as a positive control in the WD-induced hepatic steatosis mice.

Oleic acid-stimulated HepG2 cells have been widely used to evaluate NAFLD in vitro^34–39^. Consistent with in vivo experiments, our results showed that clitorin treatment significantly diminished lipid accumulation by reducing the lipogenic genes (*SREBP1*, *ACC*, *FAS*, and *HMGCR*) and enhancing the fatty acid oxidation genes (*PPARα*, *CPT-1*, and *AMPK*) in oleic acid-stimulated HepG2 cells (Fig. 5). Unexpectedly, there were significantly increases on the cell viability in 6.25–50 µM clitorin-treated cells. This issue should be considered in additional research to study the impact of clitorin in HepG2 cells, but we clearly demonstrated that 200 µM clitorin block the hepatic lipid accumulation by controlling the lipogenesis and fatty acid oxidation in oleic acid-stimulated HepG2 cells.

**Figure 6:**
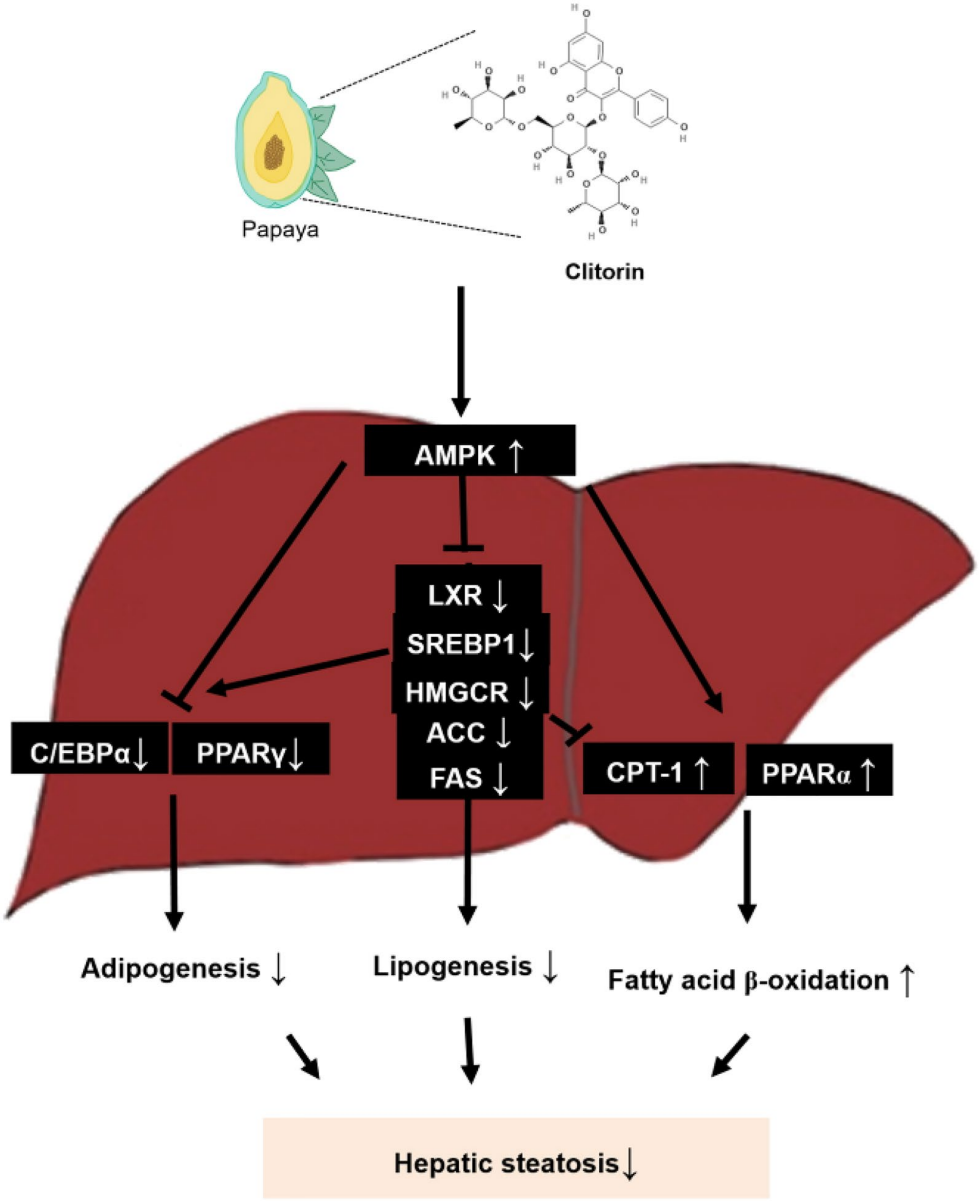
Schematic diagram of clitorin on prevention of WD-induced hepatic steatosis. Clitorin alleviated hepatic steatosis by reducing both adipogenesis and lipogenesis, and enhancing fatty acid oxidation in vivo and in vitro model.

In this study, four flavonoids, including manghaslin, clitorin, rutin, and nicotiflorin, were identified in papaya leaf. Among the four flavonoids, previous study reported that a recommended dose of rutin is 250–500 mg twice per day^40,41^. Compare to rutin, 20 mg/kg clitorin, which equates to a 97 mg for a 60 kg person, seems not to be an excessive dose for daily intake. However, it is necessary to evaluate clitorin content in papaya plant to determine whether 97 mg clitorin (for human) can be taken through diet such as papaya plant or can be considered as a food supplement. Although there is evidence on clitorin content in freeze-dried papaya leaf juice^42^, it is not sufficient to figure out its quantity. Additionally, to decide the exact human dose, further study on the toxicity of clitorin must be needed. This study contributes new knowledge to the sparse literature on clitorin, which would help specific areas for future research including determination of clinical dose. Next, we are going to analyze the mechanisms of clitorin by inhibiting/silencing AMPK in vivo and in vitro model, to fully understand its action on regulation of lipolysis and lipogenesis in NAFLD. Overall, our results showed that clitorin alleviated hepatic steatosis by reducing both adipogenesis and lipogenesis, and enhancing fatty acid oxidation (Fig. 6). The present study is the first to report on the positive impact of clitorin on hepatic steatosis and our findings provide basic data, which lead to deeper understanding of the pharmacological effects of clitorin on the potential improvement of NAFLD.

## Materials and methods

### Chemicals and reagents

Oil Red O powder, oleic acid, and methyl alcohol were purchased from Sigma-Aldrich Co. LLC (St. Louis, MO, USA). Minimum Essential Medium (MEM), fetal bovine serum (FBS), and penicillin were purchased from Life Technologies Inc. (Grand Island, NY, USA). Orlistat was purchased from Tokyo Chemical Inc. (Tokyo, Japan). The Research Diets (New Brunswick, NJ, USA) provided 45% of the WD (D-12451). Antibodies against PPARγ (cat. no. sc-7273), C/EBPα (cat. no. sc-365318), SREBP1 (cat. no. sc-13551), and β-actin (cat. No. sc-47778) were purchased from Santa Cruz Biotechnology Inc. (Dallas, TX, USA). Horseradish peroxidase-conjugated secondary antibodies were purchased from Jackson ImmunoResearch Laboratories, Inc. (West Grove, PA, USA).

### Isolation and preparation of clitorin by HPLC

Clitorin, a compound derived from *Carica papaya* L.*,* was identified by Professor Agung Nugroho (Lambung Mangkurat University, Indonesia). As previously described^43^, the leaves of *Carica papaya* were collected from a papaya farm near Pelaihari City, South Kalimantan Province, Indonesia. Plant species was identified and authenticated at the Department of Agronomy, Lambung Mangkurat University, and the voucher specimen (No. C-23) was deposited in the herbarium of Laboratory of Natural Products, Department of Agro-industrial Technology, Lambung Mangkurat University. The collected leaves were dried completely at 40 °C. The dried powder of *C. papaya* leaf (750 g) was extracted thrice with methanol (6 L) under reflux at 70 °C for 5 h. The HPLC method involved two solvents for the mobile phase, solvent A was H2O with 0.05% acetic acid, v/v) and solvent B was methanol. The linear gradient elution of the solvents was programmed as follows: 0–20 min (20 → 65% B), 20–21 min (65 → 100% B), 21–25 min (100% B), 25–27 min (100 → 20% B), and 27–30 min (20% B). The flow rate and column temperature was set constantly at 1.0 mL/min and 40 °C, respectively. The detection wavelength was fixed at 254 nm and monitored for 30 min. The linear calibration equation was y = 30.87 × + 17.49 with the LOD and LOQ was 5.91 µg/mL and 19.70 µg/mL, respectively. The purity of the compound was more than 92%. For all experiments on this plants, we confirm that all methods were carried out in accordance with relevant guidelines and regulations.

### Experimental animal care protocols and treatment cycles

Six-week-old male C57BL/6J mice were procured from Daehan Biolink (Daejeon, Republic of Korea). The mice were maintained under conditions of controlled temperature (22 ± 2 °C) and humidity (55 ± 9%), with a 12-h light/dark cycle. After a week of adjustment, the mice were fed 45% WD for 7 weeks, except for the normal diet group (CON). After 7 weeks, the mice were randomly divided into five groups of six mice each: WD group, WD + treatment group with 10 or 20 mg/kg orlistat as a positive control, and WD + treatment group with 10 or 20 mg/kg clitorin. Orlistat and clitorin were dissolved in 1:1:18 ratio of ethanol, cremophor, and distilled water and orally administered to the mice once daily for 4 weeks. Mice in the CON and WD groups were administered vehicle. The mice were allowed free access to water and food, and their body weight and food intake were measured every week. Food efficiency ratio (FER) was calculated by applying the equation: FER = (body weight gain (g)/food intake (g)) × 100. At the end of the experiment, mice were anesthetized with Zoletil 50 (20 mg/kg) by i.p. injection according to the manufacturer's instructions, and then the mice were euthanized by cervical dislocation. The livers of the mice were excised, cleaned with phosphate-buffered saline (PBS), weighed, and directly stored at − 80 °C. All experiments were performed under the Ethical Committee for Animal Care and the Use of Laboratory Animals, Sangji University (approval document no. 2017-22).

### Serum analysis

During blood sample collection, the animals were already under the influence of terminal anesthesia. Blood samples were collected via cardiac puncture. The samples were centrifuged at 1000×*g* for 20 min to obtain the serum samples. The concentrations of ALT and AST were measured by ALT and AST quantification kit (ASAN Pharm, Co., Ltd, Seoul, Republic of Korea).

### Histological analysis

The liver tissues from the mice in each group were fixed in 10% formalin, embedded in paraffin, and cut into 8 µm sections. Certain sections were stained with H&E for histological examination. Stained liver sections were observed for the evaluation of hepatic steatosis using an Olympus SZX10 microscope (Olympus, Tokyo, Japan). NAFLD development was examined using NAS, which includes a numerical score for steatosis (0–3), hepatocyte ballooning (0–2), and lobular inflammation (0–3). Steatosis were determined at 200 × magnification and quantified as macrovesicular and microvesicular steatosis using ImageJ ver. 1.50i (https://imagej.nih.gov/ij/). Then, the percentage of steatotic cells was graded as follows: (1) 0: absent; (2) 1: ≤ 25%; (3) 2: > 25% and ≤ 50%; (4) 3: > 50% and ≤ 75%; or (5) 4: > 75% of the parenchyma.

### Lipid profiling analysis

For liver tissues, approximately 0.1 g of liver tissue was homogenized in 2 mL of chloroform:methanol:distilled water (2:1:1, v/v) solution, vortexed, and centrifuged at 3,000 rpm for 10 min at room temperature. After centrifugation, the bottom layer was carefully aspirated into a new test tube and dried at 50 °C to remove chloroform. The dried lipid was weighed and dissolved in methanol prior to lipid analysis. For HepG2 cells, the supernatant from the plates was directly used. The concentrations of triglyceride and total cholesterol were measured by triglyceride and total cholesterol quantification kit (ASAN Pharm, Co., Ltd, Seoul, Republic of Korea), and detected spectrophotometrically at 550 and 500 nm.

### Western blot analysis

Each liver tissue (10 mg) was homogenized using 600 µL PRO-PREP® solution (Intron Biotechnology, Gyeonggi-do, Republic of Korea), a protein extraction solution. The same amount (15–30 µg) of protein sample was separated on an 8%–12% sodium dodecyl sulfate polyacrylamide gel and transferred onto a polyvinylidene fluoride membrane. The membranes were blocked with 2.5% skim milk solution for 30 min, incubated with PPARγ (1:1000), C/EBPα (1:1000), SREBP1 (1:1000), and β-actin (1:2500) primary antibodies overnight at 4 °C, followed by incubation with anti-mouse horseradish peroxidase-conjugated secondary antibody (1:2500) for 2 h at 25 °C. The membranes were washed thrice for 10 min with Tris-buffered saline containing Tween 20 and visualized by enhanced chemiluminescence using X-ray film (Agfa, Belgium). The uncropped blot images are given in Supplementary information.

### Quantitative reverse-transcription polymerase chain reaction (qRT-PCR) analysis

qRT-PCR analysis was performed as previously described^44^. Briefly, each liver tissue (50 mg) were homogenized, and total RNA was isolated using the 1 mL Easy-Blue® reagent according to the manufacturer’s instructions (Intron Biotechnology; Seongnam, Republic of Korea). Total RNA (1 µg) was converted to cDNA using a high-capacity cDNA reverse transcription kit (Applied Biosystems; Foster City, CA, USA). For qPCR reactions, the 10 ng of cDNA was amplified and measured using SYBR® Master Mix (Applied Biosystems; Foster City, CA, USA). Gene expression was determined using the comparative threshold cycle method. *GAPDH* was used as an internal control. Sequences of mouse oligonucleotide primers are presented in Table 1.

**Table 1:** Real-time PCR primer sequences.

Gene	Forward (5′–3′)	Reverse (5′–3′)
*PPARγ* (m)	ATCGAGTGCCGAGTCTGTGG	GCAAGGCACTTCTGAAACCG
*SREBP1* (m)	GGCTATTCCGTGAACATCTCCTA	ATCCAAGGGCAGTTCTTGTG
*C/EBPα* (m)	GGAACTTGAAGCACAATCGATC	TGGTAAAGGTTCTCA
*LXRα* (m)	CAGGAGACCAGGGAGGCAAC	GCAGGGCTGTAGGCTCTGCT
*ACC* (m)	TTTTCGATGTCCTCCCAAACTTT	GCTCATAGGCGATATAAGCTCT
*FAS* (m)	AGGGGTCGACCTGGTCCTCA	GCCATGCCCAGAGGGTGGTT
*HMGCR* (m)	CAGGATGCAGCACAGAATGT	CTTTGCATGCTCCTTGAACA
*CPT-1* (m)	CTCAGTGGGAGCGACTCTTCA	GGCCTCTGTGGTACACGACAA
*PPARα* (m)	CAGGAGAGCAGGGATTTGCA	CCTACGCTCAGCCCTCTTCAT
*AMPK* (m)	GGTGGATTCCCAAAAGTGCT	AAGCAGTGCTGGGTCACAAG
*GAPDH* (m)	ATGGAAATCCCATCACCATCTT	CGCCCCACTTGATTTTGG
*SREBP1* (h)	ACCGACATCGAAGGTGAAGT	CCAGCATAGGGTGGGTCAAA
*ACC* (h)	CATGCGGTCTATCCGTAGGTG	GTGTGACCATGACAACGAATCT
*FAS* (h)	AAGGACCTGTCTAGGTTTGATGC	TGGCTTCATAGGTGACTTCCA
*HMGCR* (h)	GACCTTTCCAGAGCAAGCAC	TCAACAAGAGCATCGAGGGT
*CPT-1* (h)	TCCAGTTGGCTTATCGTGGTG	TCCAGAGTCCGATTGATTTTTGC
*PPARα* (h)	TCCGACTCCGTCTTCTTGAT	GCCTAAGGAAACCGTTCTGTG
*AMPK* (h)	AGGATGCCTGAAAAGCTTGA	GACAGCCGGAGAAGCAGAAAC
*GAPDH* (h)	CTCCTCCACCTTTGACGCTG	CTCTTGTGCTCTTGCTGGGG

### Cell culture and treatment

The human hepatoma cell line HepG2 (No. 88065) was obtained from the Korean Cell Line Bank (KCLB, Seoul, Republic of Korea). HepG2 cells were grown in MEM containing 10% FBS and 100 mg/L penicillin under a humidified atmosphere of 5% CO_2_ at 37 °C. The cells were seeded at a density of 2 × 10^5^ cells per well into 6-well plate and then treated with 1 mM oleic acid (O 7501, Sigma-Aldrich) dissolved in culture medium containing 5% methanol with or without different concentrations of clitorin (50, 100, and 200 µM) for 48 h. The clitorin was dissolved in dimethyl sulfoxide (DMSO) and the 0.1% DMSO was treated in the control cells as a vehicle.

### Cell viability assay

HepG2 cells were seeded into a 96-well plate at a concentration of 1 × 10^4^ cells per well for 24 h. After incubation, the cells were treated with different concentrations of clitorin (0–200 µM) for 24 h. After treatment, the cells were treated with 3-(4,5-dimethylthiazol-2-yl)-2,5-diphenyl tetrazolium bromide (MTT) solution (5 mg/mL) and incubated again for 4 h. The supernatant from the plates was discarded, and the purple formazan product was dissolved in DMSO. The absorbance was measured at 540 nm using an Epoch microplate spectrometer (Biotek, Winooski, VT, USA).

### Oil red O staining of HepG2 cells

After stimulation with oleic acid, the cells were washed with PBS and fixed with 10% formaldehyde in PBS at 25 °C for 1 h. Cells were then washed thrice with distilled water and stained with Oil Red O working solution (3 mg/mL in 60% isopropanol) at 25 °C for 2 h. The cells were rinsed thrice with distilled water and photographed using an Olympus SZX10 microscope. Next, the Oil Red O dye was eluted with isopropanol to determine the intracellular lipid content and was measured using an Epoch® microvolume spectrophotometer at 520 nm.

### Statistical analysis

Data are expressed as the mean ± standard deviation (SD) of triplicate experiments. Statistically significant values were compared using ANOVA and Dunnett’s post hoc test, and *p*-values < 0.05 were considered statistically significant. Statistical analysis was performed using SPSS statistical analysis software (version 19.0, IBM SPSS, Armonk, NY, USA).

### Ethical approval

The research protocol (no. 2017-22) was approved by the Institutional animal ethics committee (IAEC) of Sangji University. Guidelines outlined in the Guide for the Care and Use of Laboratory Animals of the National Institutes of Health and the ARRIVE (Animal Research: Reporting of In-vivo Experiments) guidelines (http://www.nc3rs.org/ARRIVE) were followed to perform all the experiments.

## Supplementary Information

Supplementary Information.

## Data Availability

The datasets used and/or analyzed in this study are available from the corresponding authors on reasonable request.

## Abbreviations

AMPK: Adenosine monophosphate-activated protein kinase
ACC: Acetyl-CoA carboxylase
C/EBPα: CCAAT/enhancer binding protein α
CTP-1: Carnitine palmitoyltranserase-1
FAS: Fatty acid synthase
HMGCR: Hydroxy-3-methylglutaryl coenzyme A reductase
LXR: Liver X receptor
NAFLD: Nonalcoholic fatty liver disease
PPARα: Peroxisome proliferator-activated receptor α
PPARγ: Peroxisome proliferator-activated receptor γ
SREBP1: Sterol regulatory element-binding protein 1
WD: Western diet

## References

[1] Drescher HK, Weiskirchen S, Weiskirchen R (2019). Current status in testing for nonalcoholic fatty liver disease (NAFLD) and nonalcoholic steatohepatitis (NASH). Cells.

[2] Friedman SL, Neuschwander-Tetri BA, Rinella M, Sanyal AJ (2018). Mechanisms of NAFLD development and therapeutic strategies. Nat. Med..

[3] Pydyn N, Miekus K, Jura J, Kotlinowski J (2020). New therapeutic strategies in nonalcoholic fatty liver disease: A focus on promising drugs for nonalcoholic steatohepatitis. Pharmacol. Rep..

[4] Alves-Bezerra M, Cohen DE (2017). Triglyceride metabolism in the liver. Compr. Physiol..

[5] Lakhani HV (2018). Phenotypic alteration of hepatocytes in non-alcoholic fatty liver disease. Int. J. Med. Sci..

[6] Koo SH (2013). Nonalcoholic fatty liver disease: Molecular mechanisms for the hepatic steatosis. Clin. Mol. Hepatol..

[7] Lee YK, Park JE, Lee M, Hardwick JP (2018). Hepatic lipid homeostasis by peroxisome proliferator-activated receptor gamma 2. Liver Res..

[8] Shin MR, Shin SH, Roh SS (2020). *Diospyros kaki* and *Citrus unshiu* mixture improves disorders of lipid metabolism in nonalcoholic fatty liver disease. Can. J. Gastroenterol. Hepatol..

[9] Wang LF (2017). Inhibition of NAMPT aggravates high fat diet-induced hepatic steatosis in mice through regulating Sirt1/AMPKalpha/SREBP1 signaling pathway. Lipids Health Dis..

[10] Musso G, Gambino R, Cassader M (2009). Recent insights into hepatic lipid metabolism in non-alcoholic fatty liver disease (NAFLD). Prog. Lipid Res..

[11] Souza-Mello V (2015). Peroxisome proliferator-activated receptors as targets to treat non-alcoholic fatty liver disease. World J. Hepatol..

[12] Seo YJ, Lee K, Song JH, Chei S, Lee BY (2018). Ishige okamurae extract suppresses obesity and hepatic steatosis in high fat diet-induced obese mice. Nutrients.

[13] Pandey S, Cabot PJ, Shaw PN, Hewavitharana AK (2016). Anti-inflammatory and immunomodulatory properties of *Carica papaya*. J. Immunotoxicol..

[14] Santana LF (2019). Nutraceutical potential of *Carica papaya *in metabolic syndrome. Nutrients.

[15] Julianti T (2014). HPLC-based activity profiling for antiplasmodial compounds in the traditional Indonesian medicinal plant *Carica papaya* L. J. Ethnopharmacol..

[16] Brasil GA (2014). Antihypertensive effect of *Carica papaya* via a reduction in ACE activity and improved baroreflex. Planta Med..

[17] Ma H, Li J, An M, Gao XM, Chang YX (2018). A powerful on line ABTS(+)-CE-DAD method to screen and quantify major antioxidants for quality control of Shuxuening Injection. Sci. Rep..

[18] Hu Y (2020). Acerola polysaccharides ameliorate high-fat diet-induced non-alcoholic fatty liver disease through reduction of lipogenesis and improvement of mitochondrial functions in mice. Food Funct..

[19] Zhong F, Zhou X, Xu J, Gao L (2020). Rodent models of nonalcoholic fatty liver disease. Digestion.

[20] Ye J (2019). Effect of orlistat on liver fat content in patients with nonalcoholic fatty liver disease with obesity: Assessment using magnetic resonance imaging-derived proton density fat fraction. Therap. Adv. Gastroenterol..

[21] Noureddin M, Loomba R (2012). Nonalcoholic fatty liver disease: Indications for liver biopsy and noninvasive biomarkers. Clin. Liver Dis..

[22] Dyson JK, Anstee QM, McPherson S (2014). Non-alcoholic fatty liver disease: a practical approach to diagnosis and staging. Front. Gastroenterol..

[23] Brown GT, Kleiner DE (2016). Histopathology of nonalcoholic fatty liver disease and nonalcoholic steatohepatitis. Metabolism.

[24] Skat-Rordam J, Hojland Ipsen D, Lykkesfeldt J, Tveden-Nyborg P (2019). A role of peroxisome proliferator-activated receptor gamma in non-alcoholic fatty liver disease. Basic Clin. Pharmacol. Toxicol..

[25] Jo HK, Kim GW, Jeong KJ, Kim DY, Chung SH (2014). Eugenol ameliorates hepatic steatosis and fibrosis by down-regulating SREBP1 gene expression via AMPK-mTOR-p70S6K signaling pathway. Biol. Pharm. Bull..

[26] Shimano H (1997). Isoform 1c of sterol regulatory element binding protein is less active than isoform 1a in livers of transgenic mice and in cultured cells. J. Clin. Invest..

[27] Grønning-Wang LM, Bindesbøll C, Nebb HI (2013). The role of liver X receptor in hepatic de novo lipogenesis and cross-talk with insulin and glucose signaling. Lipid Metab..

[28] Kohjima M (2008). SREBP-1c, regulated by the insulin and AMPK signaling pathways, plays a role in nonalcoholic fatty liver disease. Int. J. Mol. Med..

[29] Montagner A (2016). Liver PPARalpha is crucial for whole-body fatty acid homeostasis and is protective against NAFLD. Gut.

[30] Pawlak M, Lefebvre P, Staels B (2015). Molecular mechanism of PPARalpha action and its impact on lipid metabolism, inflammation and fibrosis in non-alcoholic fatty liver disease. J. Hepatol..

[31] Reddy JK, Rao MS (2006). Lipid metabolism and liver inflammation. II. Fatty liver disease and fatty acid oxidation. Am. J. Physiol. Gastrointest. Liver Physiol..

[32] Liou CJ (2018). Fisetin protects against hepatic steatosis through regulation of the Sirt1/AMPK and fatty acid beta-oxidation signaling pathway in high-fat diet-induced obese mice. Cell Physiol. Biochem..

[33] Inamdar S, Joshi A, Malik S, Boppana R, Ghaskadbi S (2019). Vitexin alleviates non-alcoholic fatty liver disease by activating AMPK in high fat diet fed mice. Biochem. Biophys. Res. Commun..

[34] Kanuri G, Bergheim I (2013). In vitro and in vivo models of non-alcoholic fatty liver disease (NAFLD). Int. J. Mol. Sci..

[35] Müller FA, Sturla SJ (2019). Human in vitro models of nonalcoholic fatty liver disease. Curr. Opin. Toxicol..

[36] Lee MR, Yang HJ, Park KI, Ma JY (2019). Lycopus lucidus Turcz. ex Benth Attenuates free fatty acid-induced steatosis in HepG2 cells and non-alcoholic fatty liver disease in high-fat diet-induced obese mice. Phytomedicine.

[37] Ali O, Darwish HA, Eldeib KM, Abdel Azim SA (2018). miR-26a potentially contributes to the regulation of fatty acid and sterol metabolism in vitro human HepG2 cell model of nonalcoholic fatty liver disease. Oxid. Med. Cell Longev..

[38] Gomaraschi M (2019). Lipid accumulation impairs lysosomal acid lipase activity in hepatocytes: Evidence in NAFLD patients and cell cultures. Biochim. Biophys. Acta Mol. Cell. Biol. Lipids.

[39] Xia H (2019). Alpha-naphthoflavone attenuates non-alcoholic fatty liver disease in oleic acid-treated HepG2 hepatocytes and in high fat diet-fed mice. Biomed. Pharmacother..

[40] Yu CP (2011). Quercetin and rutin reduced the bioavailability of cyclosporine from Neoral, an immunosuppressant, through activating P-glycoprotein and CYP 3A4. J. Agric. Food Chem..

[41] Volate SR, Davenport DM, Muga SJ, Wargovich MJ (2005). Modulation of aberrant crypt foci and apoptosis by dietary herbal supplements (quercetin, curcumin, silymarin, ginseng and rutin). Carcinogenesis.

[42] Mohd Abd Razak MR (2021). Immunomodulatory activities of *Carica papaya* L. leaf juice in a non-lethal symptomatic dengue mouse model. Pathogens.

[43] Nugroho A, Heryani H, Choi JS, Park H-J (2017). Identification and quantification of flavonoids in *Carica papaya* leaf and peroxynitrite-scavenging activity. Asian Pac. J. Trop. Biomed..

[44] Park YJ, Lee GS, Cheon SY, Cha YY, An HJ (2019). The anti-obesity effects of Tongbi-san in a high-fat diet-induced obese mouse model. BMC Complement. Altern. Med..

