# Supplementary material for: Clitorin ameliorates western diet-induced hepatic steatosis by regulating lipogenesis and fatty acid oxidation in vivo and in vitro
Source: Sci Rep. 2022 Mar 9;12:4154. doi: 10.1038/s41598-022-07937-3 (PMC8907210; doi:10.1038/s41598-022-07937-3)
Supplement: Supplementary file 1 — Supplementary Information. [file 41598_2022_7937_MOESM1_ESM.pdf]

Fig. 4A

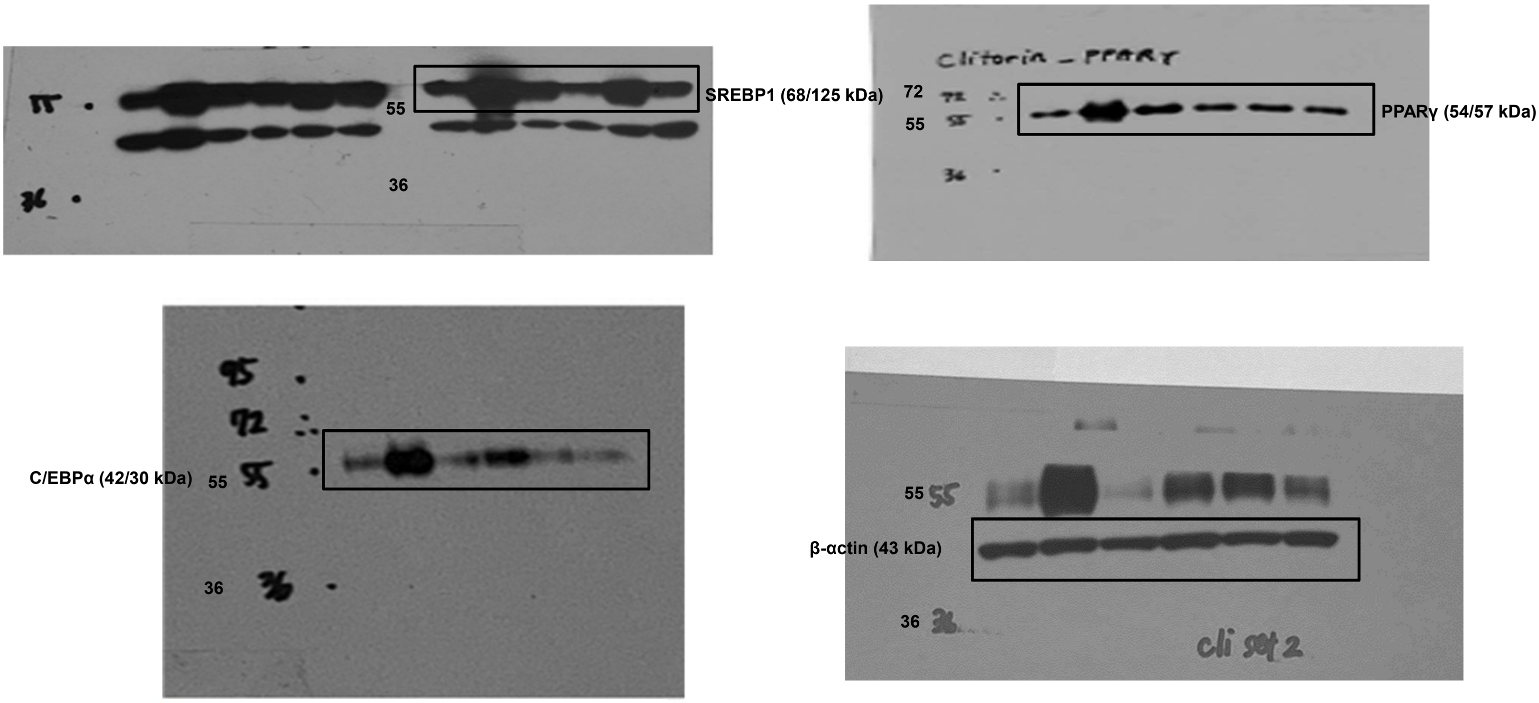

Molecular Weight of C/EBP α isoforms: 42/30 kDa.

Positive Controls: Caki-1 cell lysate: sc-2224, HeLa whole cell lysate: sc-2200 or rat liver extract: sc-2395.

DATA

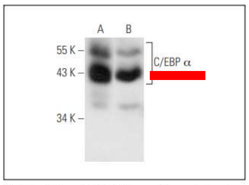

C/EBP α (D-5): sc-365318. Western blot analysis of C/EBP α expression in Caki-1 (**A**) and HeLa (**B**) whole cell lysates.
